# Effects of Pomegranate Extract on IGF-1 Levels and Telomere Length in Older Adults (55–70 Years): Findings from a Randomised Double-Blinded Controlled Trial

**DOI:** 10.3390/nu17182974

**Published:** 2025-09-16

**Authors:** Grace Farhat, Jhama Malla, Liam Hanson, Jay Vadher, Emad A. S. Al-Dujaili

**Affiliations:** 1 Faculty of Health and Education, Manchester Metropolitan University, Manchester M15 6GX, UK; 2 Faculty of Health and Medical Sciences, University of Surrey, Guildford GU2 7YH, UK; 3 Faculty of science and Engineering, Manchester Metropolitan University, Manchester M15 5GD, UK; l.hanson@mmu.ac.uk; 4 Faculty of Sport and Exercise, Manchester Metropolitan University, Manchester M15 6BH, UK; j.vadher@mmu.ac.uk; 5 Centre for Cardiovascular Science, Faculty of Medicine and Veterinary Medicine, Queen’s Medical Research Institute, University of Edinburgh, Edinburgh EH16 4TJ, UK; ealduja1@exseed.ed.ac.uk

**Keywords:** pomegranate extract, polyphenols, ageing, telomere length, IGF-1

## Abstract

**Background**: Emerging evidence suggests that polyphenols may contribute to the attenuation of telomere attrition and the upregulation of insulin-like growth factor 1 (IGF-1), primarily in animal and cell studies, and to a lesser extent in humans. Pomegranate extract, known for its high antioxidant capacity, has shown promise in preventing telomere shortening and enhancing IGF-1 levels, but evidence in humans is lacking. **Objective**: To investigate the effects of pomegranate extract on telomere length and serum IGF-1 levels in older adults aged 55–70 years. **Methods**: Participants took part in a two-arm double-blind parallel trial, receiving either placebo capsules (maltodextrin) or pomegranate extract (740 mg) daily for 12 weeks. At baseline, week 6 and week 12, anthropometric measurements, blood pressure readings and blood samples were collected. Telomere length and serum IGF-1 levels were assessed. **Results**: A total of 72 participants completed the study. Analysis showed a significant effect of treatment and time on IGF-1 ((F_2,136_ = 3.43, *p* = 0.04), with levels significantly increasing in the pomegranate extract group at week 12. No significant effects on telomere length were noted. Weight status, physical activity, age, gender and energy intake did not impact the outcomes. **Conclusions**: Pomegranate extract significantly increased IGF-1 levels and could exert a positive role on vascular ageing. Further research is needed to replicate these findings and confirm its long-term benefits. Extended studies are required to elucidate its potential to counteract telomere shortening.

## 1. Introduction

With the global population of older adults steadily increasing, there is a corresponding increase in age-related diseases and health challenges [1]. Ageing is a complex biological process influenced by genetic, environmental and lifestyle factors, and multiple factors are involved in the manifestation of ageing, including telomere shortening [2]. Telomeres are protective caps at the end of chromosomes that prevent their instability by shielding genetic material from degradation, preventing chromosomal end-to-end fusions and ensuring proper replication during cell division. As telomeres progressively shorten with each cell division, they contribute to cellular ageing and senescence [3]. It has been suggested that telomere length measurement could serve as a promising clinical tool for age-related diseases [4], and there is evidence showing an inverse association between telomere length and BMI [5], as well as between telomere length and blood pressure levels [6]. Evidence from cell research showed that oxidative stress can accelerate telomere shortening, thereby speeding the ageing process and contributing to age-related diseases [7]. Targeting oxidative stress could therefore potentially represent a therapeutic strategy to slow down the ageing process and age-related diseases.

IGF-1 is a hormone increasingly recognised for its role in ageing and longevity, though its role remains controversial. Multiple studies suggest that IGF-1 is a biological marker of ageing, with lower concentrations associated with an increased risk of atherosclerosis, cardiovascular mortality and heart failure in older adults [8,9]. Findings from a review including both animal and human studies additionally highlight the role of IGF-1 in reducing oxidative stress, apoptosis and inflammatory signalling, suggesting a potential disease protective role in normal vascular ageing [10,11,12]. Although the exact mechanisms remain unclear, it is hypothesized that the effect may be mediated through the downregulation of tumour necrosis factor-alpha (TNF-α) in macrophages, an effect reported in humans [11]. However, IGF-1 has also been associated with negative effects on longevity, with proposed mechanisms including increased cellular proliferation and risk of tumour [13].

Some evidence suggests a complex interaction between IGF-1 and telomere length, proposing that elevated IGF-1 levels can independently predict longer telomere length. A study including 551 adults aged 65 years and older reported a significant association between higher IGF-1 and longer telomere length after adjusting for age, gender, disease status and BMI [14]. IGF-1 may reduce oxidative stress and inflammation [15], which are thought to be key mechanisms driving increased immune cell turnover and telomere shortening.

Polyphenols, abundant in fruits and vegetables, are well-known for their antioxidant and anti-inflammatory properties and have garnered increasing interest for their potential anti-ageing effects. Limited human studies suggest that polyphenols may reduce telomere shortening, likely due to their antioxidant and anti-inflammatory activities [16,17,18]. Furthermore, polyphenols have been shown to upregulate IGF-1 in both animal and cell models [19,20]. Pomegranate extract (PE) has recently attracted particular attention due to its potent antioxidant properties and its potential as a sustainable option in the face of climate change [21,22]. An animal study demonstrated that administering pomegranate peel for two months enhanced telomerase reverse transcriptase expression, reduced oxidative stress and elevated IGF-1 levels in aged rats [23]. Given the limited studies, our study aimed to explore the effects of PE on telomere length and IGF-1 levels in older adults (55–70 years), with the goal of gaining insights into the mechanisms linking polyphenols to ageing and informing the need for longer-term studies.

## 2. Materials and Methods

The study was conducted according to the guidelines laid down in the Declaration of Helsinki and received ethical approval from the Manchester Metropolitan University Faculty of Health and Education (reference number: 47627). Written informed consent was obtained from all participants before they joined the study. Recruitment occurred between December 2022 and June 2024. The study was registered with clinicaltrials.gov (NCT05588479).

### 2.1. Intervention

The protocol of the study has been described elsewhere [24,25]. In brief, this was a two-arm double-blind parallel trial, where participants were randomly assigned to receive either placebo (PL) capsules (maltodextrin) or PE capsules (two 370 mg capsules of PE each) daily for 12 weeks. Each PE capsule contained 36% punicalagins and 1.3% ellagic acid, with the remaining content composed primarily of maltodextrin, other polyphenols and nutrients naturally occurring in the fruits, such as carbohydrates. The PE was tested to ensure it was free from pesticide residues, heavy metals, aflatoxins and microbiological contamination. Random assignment was computer-generated, and groups were matched for gender and BMI. Recruitment staff who were blinded to the intervention groups conducted this process to minimise selection bias. The pomegranate and placebo capsules looked similar and were distributed to participants in two batches at baseline and week 6.

Participants visited the Physiology Lab at baseline, week 6, and week 12. During each visit, anthropometric measurements were taken, including weight (Marsden DP2400 digital scale, London, UK), height (Seca^®^ 711, Hamburg, Germany), waist and hip circumferences (elastic measuring tape) and body composition (air displacement plethysmography with a Bodpod^®^: Cosmed GS-X, Rome, Italy). Blood pressure was measured three times after a 10 min rest period, following the WHO protocol [26] using a digital sphygmomanometer (Nissei^®^ DS-1873, Tokyo, Japan). A 20 mL fasted venous blood sample was also collected, processed and stored at −80 °C until analysis. Participants completed a socio-demographic questionnaire before or during the first visit, providing information on age, gender, occupation, ethnicity and physical activity. Physical activity levels were expressed in METs (Metabolic Equivalent of Tasks), which indicate the energy expenditure of each activity relative to resting. The MET values were estimated using widely available guidelines for different activities [27].

#### 2.1.1. Participants

Recruitment, inclusion and exclusion criteria were mentioned elsewhere [24,25]. Volunteers were recruited through various channels, including posters, community centres, events, gyms, university premises and social media. Eligibility was screened online or in person, and appointments were scheduled at the Physiology Lab at Manchester Metropolitan University, UK. Participants fasted for at least 8 h before each appointment. Inclusion criteria were English-speaking adults aged 55–70 years with a BMI of 18.5–29.9 kg/m^2^. Obese individuals were excluded to keep the study’s focus on prevention. Exclusion criteria included recent weight loss regimens, chronic diseases and medications affecting blood pressure, lipid levels or inflammation.

#### 2.1.2. Laboratory Analysis

IGF-1 was quantified using the Human IGF-I/IGF-1 ELISA Kit (Bio-Techne^®^, Minneapolis, MN, USA, protocol DG100B). In brief, serum samples were defrosted, centrifuged and diluted (1:90) according to protocol. The plate was first washed twice with wash buffer. Subsequently, 50 µL of Assay Diluent RD1-99 were added to each well, followed by 50 µL of standard, control or sample. The plate was incubated for 3 h at 4 °C. The wells were then washed four times, and 200 µL of Human IGF-1 Conjugate was added to each well. After another incubation for 1 h at room temperature and a subsequent wash, 200 µL of substrate solution was added, and the plate was incubated for 30 min at room temperature. Finally, 50 µL of stop solution was added to each well, and the optical density was measured at 450 nm.

Telomeres were measured using a combination of droplet digital PCR (Bio-rad^®^, Livermore, CA, USA, QX200) and quantitative PCR (Bio-Rad^®^, Livermore, CA, USA, CFX Connect) with telomere length determined by ddPCR and gene copy number confirmed using qPCR. The primers used were based of those previously used by O’Callaghn et al. [28] and detailed in Table 1. Telomere length was determined using ddPCR generating a standard curve (Table 2) to calculate base pairs of telomeres within samples with data normalised to an average non-template control (NTC value). The Kilobase (Kb) of telomeres within the standards were calculated using the methodology described (28), while the following equation was used to determine the Kb of telomeres from the ddPCR results:

$$\frac{Copy\;no.\;per\;uL\times Reaction\;Volume}{Template\;Volume}$$

To control for changes in genome copies within samples, copy numbers of a targeted gene, 36B4, were determined by qPCR, generating a standard curve and using known standards before calculating the number of diploid genome copies within each sample (Table 3). The Kb of telomere per sample was then adjusted using the gene copy number to determine the Kb or telomere per diploid genome.

#### 2.1.3. Compliance

Participants were given a paper-based diary to log the dates of their capsule intake, marking each entry with a checkmark. During each appointment, they were asked about their consistency in taking the capsules daily, and their responses were noted. Compliance was calculated for each participant based on the number of capsules they reported missing during the intervention period. The percentage of capsules taken was then averaged across all participants to determine overall compliance. To track any changes in dietary intake, participants were instructed to complete a 3-day diet diary (covering 2 weekdays and 1 weekend) at three points during the intervention: baseline, week 6, and week 12.

#### 2.1.4. Power Calculation and Statistical Analysis

A sample size of 84 participants was determined based on the primary outcome of the study (decrease in IL-6 levels) [23]. However, we determined that a sample size of 22 participants per group is sufficient to detect a 25% difference in telomere length between groups, with 95% power and a 0.05 error rate using repeated-measures ANOVA within–between interaction. Sample size calculation was calculated using G*Power software version 3.1.9.7. This data was derived from the study by Tran et al. (2019) [17].

Data were analysed using SPSS version 29 (IBM, Chicago, IL, USA) and presented as mean (SD), unless otherwise noted as standard error (SE). Normality was assessed using the Kolmogorov–Smirnov test. For categorical variables, baseline differences were assessed using the Chi-square exact test. Repeated-measures ANOVA was used to assess the interaction between treatment (PE vs. PL) and time (baseline, week 6 & week 12). Pairwise comparisons were conducted using the Bonferroni test to identify significant differences. The impact of variables (e.g., age, gender, energy intake and physical activity levels) on the outcomes was assessed using repeated-measures ANOVA with the variables as covariates. Correlation between different outcome parameters was assessed using Pearson’s correlation coefficient. Statistical significance was defined at *p* ≤ 0.05.

## 3. Results

**Figure 1:**
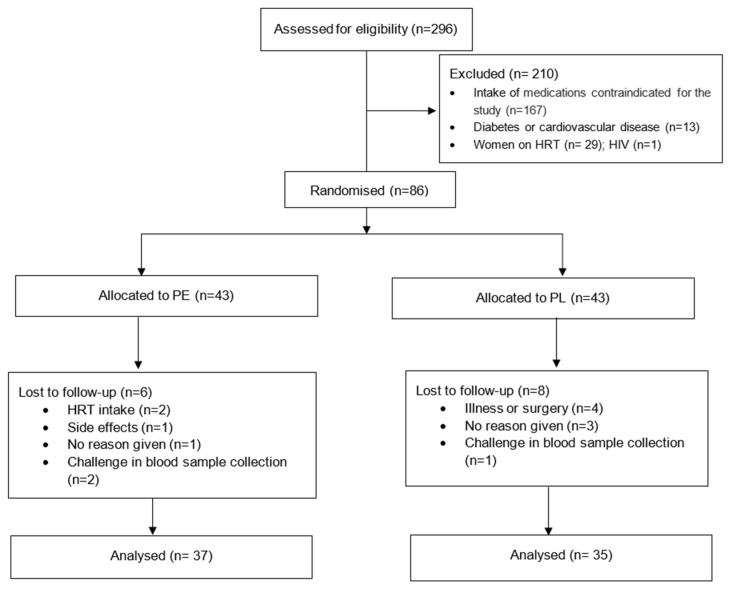
Consort flow diagram. Abbreviations: PE: pomegranate extract; PL: placebo; HRT: hormone replacement therapy.

The advertisement led to 355 individuals expressing interest, of whom 296 were assessed for eligibility and 86 met the eligibility criteria for participation. These participants were equally assigned to the PE and PL groups. Eight participants withdrew after their initial appointment, leaving 76 who completed the full intervention. In some cases (n = 4), challenges with blood collection during specific appointments resulted in incomplete data for certain participants. Consequently, 72 participants completed data from all three appointments and were included in the final analysis. The overall attrition rate was 11.6%. The CONSORT flow diagram has included in Figure 1.

The majority of participants were female (61%) and White British (83%). The mean age of the population is 61.22 (4.31) years, and the average BMI was 23.91 (3.25) kg/m^2^, with 65.28% belonging to the normal weight category. Characteristics of the study population is presented in Table 4.

Independent *t*-tests showed no significant differences in age (*p* = 0.12) and baseline levels of BMI (*p* = 0.78), waist circumference (*p*= 0.31), waist-to-hip ratio (*p* = 0.3), body fat percentage (*p* = 0.58), SBP (*p* = 0.71) and DBP (*p* = 0.52) between the PE and PL groups. No significant between-group differences in gender (*p*= 0.85) and ethnicity (*p* = 0.33) were noted. Compliance was estimated to be high (87%).

### 3.1. Effects of Pomegranate Extract on IGF-1 Levels

**Figure 2:**
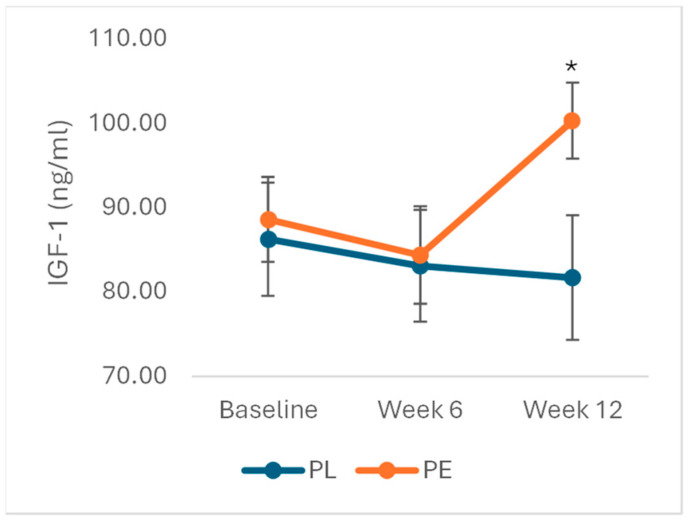
Effects of PE on IGF-1 levels. Data is expressed as mean (SE) and analysed using repeated measures ANOVA. In the PE group (n = 35), IGF-1 increased by 14.09 (6.87) SE ng/mL (*p* = 0.04) at week 12, while changes were not significant in the PL group (n = 35) (4.56 (4.97) SE ng/mL, *p* = 0.37). * *p* < 0.05. Abbreviations: PE: pomegranate extract; PL: placebo; IGF-1: insulin growth factor-1.

After excluding outliers (n = 2), data was normally distributed. Analysis showed a significant effect of treatment and time on IGF-1 levels (F_2,136_ = 3.43, *p* = 0.04). In the PE group, IGF-1 levels increased significantly by 14.09 ng/mL (SE = 6.87, *p* = 0.02) at week 12, with no significant changes observed at week 6. No significant changes were detected in PL group at either time point (Figure 2).

### 3.2. Effects of PE on Telomere Length

**Figure 3:**
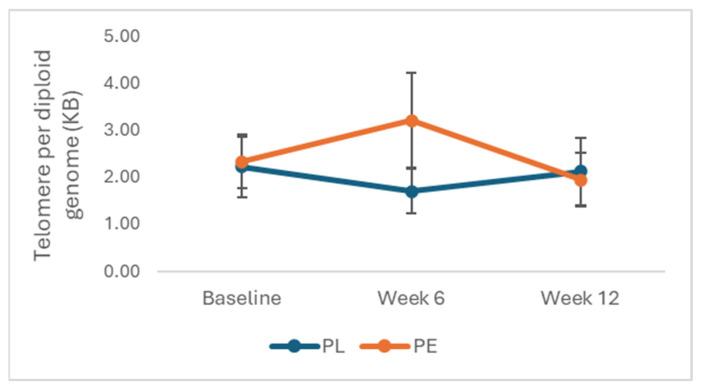
Effects of PE on telomere length. Data is expressed as mean (SE) and analysed using repeated measures ANOVA after logarithm transformation of data. Abbreviations: PE: pomegranate extract; PL: placebo.

Analysis showed no significant effect of treatment and time on telomere length (F_2,128_ = 0.41, *p* = 0.66) following 12 weeks of intervention (Figure 3). Additionally, Pearson’s correlation showed no significant interaction between telomere length and IGF-1 levels (*p* = 0.5). Further analysis showed no association between telomere length and baseline levels of BMI (*p* = 0.34), SBP (*p* = 0.88) or DBP (*p* = 0.61).

### 3.3. Changes in Energy Intake and MET Levels

MET levels did not differ between groups at the end of the intervention (F_1,12_= 0.12, *p* = 0.7). Additionally, analysis of diet diaries showed no significant differences in energy (F_1.7,107_ = 0.11, *p* = 0.86), carbohydrate (F_1.8, 112_ = 0.19, *p*= 0.8) protein (F_1.14,72_ = 0.37) or fat intake (F_1.8,114_ = 0.2, *p* = 0.8) between the PE and PL groups at the end of intervention. Data at baseline, week 6 and week 12 are added to Supplementary Material S1.

### 3.4. Impact of Confounding Factors on the Outcomes

Analysis for IGF-1 levels and telomere length were repeated while using age, baseline physical activity levels (MET) and energy intake as covariates while gender was used as a fixed factor. Results showed no significant impact of these outcomes on changes in IGF-1 levels (F_2,54_ = 1.23, *p* = 0.3) or telomere levels (F_2,46_ = 0.77, *p =* 0.47).

## 4. Discussion

This study aimed to explore the effects of PE on telomere length and IGF-1 levels in older adults. While telomere length did not significantly change after 12 weeks of intervention, IGF-1 levels showed a small but statistically significant increase (*p* = 0.04). These findings are important, as they contribute to ongoing efforts to understand the effects of plant-based extracts, particularly PE as modulators of age-related biological processes and promoters of healthy ageing.

The complex role of IGF-1 in ageing suggests that these results need to be considered in context. IGF-1 has been reported to have a positive role in cardiovascular protection, exhibit insulin-like activity at higher concentrations and play a key role in bone metabolism and muscle regeneration [29]. Declining IGF-1 levels with age have been associated with reduced muscle mass [30], increased oxidative stress [31] and decreased bone mineral density [32], highlighting its importance in tissue repair and anabolic maintenance. However, elevated IGF-1 activity has also been linked to increased cancer risk and reduced lifespan, suggesting that while it supports tissue maintenance, excessive or dysregulated IGF-1 signalling may negatively impact healthy ageing [29].

In this study, the significant increase in IGF-1 levels is consistent with previous findings from animal and cellular models, where polyphenol administration was shown to upregulate IGF-1 expression [19,20,23]. Pomegranate has also been reported to improve muscle strength and recovery [33,34], effects that may be mediated through enhanced IGF-1 signalling and attenuation of oxidative stress [23]. One proposed mechanism is that polyphenols in PE stimulate hepatic IGF-1 production, thereby increasing systemic availability [35] which may contribute to improved musculoskeletal health in older adults, alongside a decrease in oxidative stress. We suggest that the modest increase observed in this study (14.09 ± 6.87 ng/mL) may be beneficial without posing adverse effects, although this hypothesis requires further investigation in long-term human studies.

The lack of significant change in telomere length indicates that this intervention did not counteract the shortening of telomeres in this older population. These results contrast with previous animal studies on the effects of pomegranate on telomeres [23] and other human studies involving different types of polyphenols [36,37]. This discrepancy may be attributed to several factors, including intervention duration, age range and baseline health status of participants. Human studies involving older populations are complex due to several influences, including the presence of various risk factors, existing health conditions and age-related declines in metabolism, which affect the bioavailability and metabolism of polyphenols [38]. Our study population was physically active and free from chronic disease, which may have influenced the outcomes by reducing the potential for observable improvements in telomere length. Notably, telomere length is a relatively stable biomarker that typically changes gradually over long periods, even years [39]. Therefore, the 12-week duration of our study may have been insufficient to detect measurable changes in telomere length. Future research should consider longer-term interventions, as well as stratifying participants by age and health status. Additionally, some studies have reported significant changes in telomerase activity rather than telomere length [36], which may highlight a methodological limitation in our study. Measuring telomerase activity could provide a more sensitive indicator of short-term effects in telomere. Finally, a larger sample size may be necessary to detect subtle changes in telomere length and to improve the statistical power of future investigations.

Furthermore, the relatively short duration of the intervention (12 weeks) may have been insufficient to observe any potential interaction between telomere and IGF-1. Telomere typically changes over longer periods, and transient changes in IGF-1 may not immediately translate into measurable effects on telomere length.

This is the first study to assess the effect of PE on telomere length in humans, focusing on older adults and examining the impact of multiple confounding factors on the outcomes. Nonetheless, several limitations should be acknowledged. Firstly, the relatively short duration of the intervention (12 weeks) may have been insufficient to detect measurable changes in telomere length, which typically occur over extended periods. Secondly, the sample size may still have lacked the statistical power to identify small but potentially meaningful effects. Thirdly, the predominance of participants with a normal body weight does not reflect the broader population, potentially affecting generalisability of results. Furthermore, the study did not assess telomerase activity, which could have provided further insights into the underlying biological mechanisms. Lastly, although dietary intake was recorded, polyphenol consumption was not specifically analysed. Given that certain polyphenols may influence IGF-1 levels, we cannot exclude the possibility that background dietary polyphenol intake may have contributed to the observed effects. Future studies should consider using biomarkers to better control for this potential confounder. These limitations highlight the need for longer-term, larger-scale studies to more comprehensively evaluate the impact of PE on telomere length and biological ageing.

## 5. Conclusions

The findings of this study suggest that daily supplementation with 740 mg of PE may elevate IGF-1 levels, indicating its potential to support healthy ageing in older adults, although the broader implications for longevity remain to be clarified. Future research should focus on long-term, large-scale studies to confirm these effects and further investigate the potential long-term effects of PE on counteracting telomere shortening and contributing to improved lifespan.

## Figures and Tables

**Table 1:** Primer Sequences.

Target	Sequencer
Telomere Forward Primer	CGGTTTGTTTGGGTTTGGGTTTGGGTTTGGG TTTGGGTT
Telomere Reverse Primer	GGCTTGCCTTACCCTTACCCTTACCC TTACCCTTACCCT
Telomere Standard	(TTAGGG)14
36B4 Forward Primer	CAGCAAGTGGGAAGGTGTAATCC
36B4 Reverse Primer	CCCATTCTATCATCAACGGGTACAA
36B4 Standard	CAGCAAGTGGGAAGGTGTAATCCGTCTCCACAGACAAGGCCAGGACTCGTTTGTACCCGTTGATGATAGAATGGG

**Table 2:** (**a**) ddPCR Reaction Components, (**b**) ddPCR Cycling Parameters (**c**) Telomere Standards.

(**a**)		
0.25 ng of template DNA/Standard		
100 nM forward primer		
100 nM Reverse primer		
(**b**)		
Reaction Stage	Temperature	Time
Enzyme Activation	95 °C	5 min
Denaturation	95 °C	30 s (40 cycles)
Annealing/Extension	53 °C	1 min (40 cycles)
Signal Stabilization 1	4 °C	5 min
Signal Stabilization 2	90 °C	5 min
Hold	4 °C	infinite
(**c**)		
Standard	Quantity, pg^−μL^	Kb of Telomere
A	1.875	7.38 × 10^6^
B	0.938	3.69 × 10^6^
C	0.469	1.84 × 10^6^
D	0.234	9.22 × 10^5^
E	0.117	4.61 × 10^5^
F	0.059	2.30 × 10^5^
G	0.029	1.15 × 10^5^

Abbreviations: DNA: Deoxyribonucleic Acid; EVA: Ethylene-Vinyl Acetate; ddPCR: Droplet Digital Polymerase Chain Reaction; RNase/DNase: Ribonuclease/Deoxyribonuclease.

**Table 3:** (**a**) qPCR Reaction Components—40 reaction (**b**) qPCR Cycling Parameters (**c**) 36B4 Standards.

(**a**)		
60 ng of template DNA/Standard		
100 nM forward primer		
100 nM Reverse primer		
2× Quantinova SYBR Green Mastermix		
RNase/DNase free water		
(**b**)		
Reaction Stage	Temperature	Time
Denaturation	95 °C	10 min
Denaturation	95 °C	15 s (40 cycles)
Annealing/Extension	60 °C	1 min (40 cycles)
Dissociation Curve		
Denaturation	95 °C	10 s
Dissociation	60–95 °C	25 min
(**c**)		
Standard	Quantity, pg^−μL^	Diploid Copies
A	200	2.63 × 10^9^
B	20	2.63 × 10^8^
C	2	2.63 × 10^7^
D	0.2	2.63 × 10^6^
E	0.02	2.63 × 10^5^
F	0.002	2.63 × 10^4^
G	0.0002	2.63 × 10^3^

Abbreviations: qPCR: Quantitative Polymerase Chain Reaction; RNase/DNase: Ribonuclease/Deoxyribonuclease; DNA: Deoxyribonucleic Acid; SYBR: SYBR Green dye used for DNA detection in qPCR.

**Table 4:** Baseline characteristics of the intention-to-treat population by intervention group.

Characteristics	All Participants (n = 72)	PE Group (n = 37)	PL Group (n = 35)
Age at baseline, y	61.22 (4.31)	60.46 (3.90)	62.03 (4.62)
Sex (n)			
Female	44	23	21
Male	28	14	14
Occupation category (n)			
Professional Occupations	32	13	19
Managers, Directors & Senior Officials	6	3	3
Health-related Professions	5	1	4
Associate Professional &Technical Occupations	3	1	2
Administrative & Secretarial Occupations	4	4	0
Unemployed	2	2	0
Sales & Customer Service Occupations	2	2	0
Caring, Leisure & Other Service Occupations	1	1	0
Retired	17	7	10
Ethnicity (n)			
White British	60	32	28
White Irish	3	1	2
Indian	1	0	1
Other white	2	0	2
Mixed race	2	2	0
Black	1	0	1
Other	3	2	1
Smoking status (n)			
Smoker	3	1	2
Non-smoker	69	36	35
MET (MET-minutes/week)	731 (72)	742 (33)	727 (45)
BMI (kg/m^2^)	23.91 (3.25)	23.80 (3.20)	24.01 (3.34)
Waist circumference (cm)	84.75 (10.28)	83.54 (9.82)	86.04 (10/73)
Waist-to-hip ratio	0.84 (0.07)	0.83 (0.07)	0.85 (0.07)
Body fat percentage (%)	22.03 (10.02)	22.68 (9.43)	21.34 (10.69)
SBP (mmHg)	128.97 (12.48)	128.43 (13.57)	129.54 (11.38)
DBP (mmHg)	81.38 (8.60)	80.74 (9.54)	82.06 (7.55)

Values are presented as mean (SD). Baseline differences between groups were analysed using an independent *t*-test for numerical variables and Chi square test for nominal variables. No significant differences between groups were observed (*p* > 0.05). Abbreviations: PE: Pomegranate extract; PL: Placebo; SBP: Systolic blood pressure; DBP: Diastolic blood pressure; MET: Metabolic Equivalent of Task; BMI: Body Mass Index.

## Data Availability

The data presented in the manuscript will not be made available, as it is needed for ongoing research and analysis.

## Supplementary Materials

The following supporting information can be downloaded at: https://www.mdpi.com/article/10.3390/nu17182974/s1, Supplementary Material S1: Changes in Energy and macronutrient intake across three time points.

## Abbreviations

The following abbreviations are used in this manuscript:

PE	Pomegranate extract
PL	Placebo
IGF-1	Insulin growth Factor-1
SBP	Systolic blood pressure
DBP	Diastolic blood pressure
TNF-α	Tumour necrosis factor-alpha

## References

[1] WHO Ageing and Health. https://www.who.int/news-room/fact-sheets/detail/ageing-and-health.

[2] Rodríguez-Rodero S., Fernández-Morera J.L., Menéndez-Torre E., Calvanese V., Fernández A.F., Fraga M.F. (2011). Aging genetics and aging. Aging Dis..

[3] O’sullivan R.J., Karlseder J. (2010). Telomeres: Protecting chromosomes against genome instability. Nat. Rev. Mol. Cell Biol..

[4] Hsieh A.Y., Saberi S., Ajaykumar A., Hukezalie K., Gadawski I., Sattha B., Côté H.C. (2016). Optimization of a relative telomere length assay by monochromatic multiplex real-time quantitative PCR on the LightCycler 480: Sources of variability and quality control considerations. J. Mol. Diagn..

[5] An R., Yan H. (2017). Body weight status and telomere length in US middle-aged and older adults. Obes. Res. Clin. Pract..

[6] Huang Y.Q., Shen G., Lo K., Huang J.Y., Liu L., Yu Y.L., Chen C.L., Zhang B., Feng Y.Q. (2019). The Relationship between Mean Telomere Length and Blood Pressure: Results from the National Health and Education National Surveys. SSRN.

[7] Von Zglinicki T. (2002). Oxidative stress shortens telomeres. Trends Biochem. Sci..

[8] Laughlin G.A., Barrett-Connor E., Criqui M.H., Kritz-Silverstein D. (2004). The prospective association of serum insulin-like growth factor I (IGF-I) and IGF-binding protein-1 levels with all cause and cardiovascular disease mortality in older adults: The Rancho Bernardo Study. J. Clin. Endocrinol. Metab..

[9] Vasan R.S., Sullivan L.M., D’Agostino R.B., Roubenoff R., Harris T., Sawyer D.B., Levy D., Wilson P.W. (2003). Serum insulin-like growth factor I and risk for heart failure in elderly individuals without a previous myocardial infarction: The Framingham Heart Study. Ann. Intern. Med..

[10] Higashi Y., Sukhanov S., Anwar A., Shai S.Y., Delafontaine P. (2010). IGF-1, oxidative stress and atheroprotection. Trends Endocrinol. Metab..

[11] Serri O., Li L., Maingrette F., Jaffry N., Renier G. (2004). Enhanced lipoprotein lipase secretion and foam cell formation by macrophages of patients with growth hormone deficiency: Possible contribution to increased risk of atherogenesis?. J. Clin. Endocrinol. Metab..

[12] Sukhanov S., Higashi Y., Shai S.Y., Vaughn C., Mohler J., Li Y., Song Y.H., Titterington J., Delafontaine P. (2007). IGF-1 reduces inflammatory responses, suppresses oxidative stress, and decreases atherosclerosis progression in ApoE-deficient mice. Arterioscler. Thromb. Vasc. Biol..

[13] Fontana L., Partridge L., Longo V.D. (2010). Extending healthy life span—From yeast to humans. Science.

[14] Kaplan R.C., Fitzpatrick A.L., Pollak M.N., Gardner J.P., Jenny N.S., McGinn A.P., Kuller L.H., Strickler H.D., Kimura M., Psaty B.M. (2009). Insulin-like growth factors and leukocyte telomere length: The cardiovascular health study. J. Gerontol. Ser. A Biomed. Sci. Med. Sci..

[15] Rajpathak S.N., McGinn A.P., Strickler H.D., Rohan T.E., Pollak M., Cappola A.R., Kuller L., Xue X., Newman A.B., Strotmeyer E.S. (2008). Insulin-like growth factor-(IGF)-axis, inflammation, and glucose intolerance among older adults. Growth Horm. IGF Res..

[16] Pereira Q.C., Dos Santos T.W., Fortunato I.M., Ribeiro M.L. (2023). The molecular mechanism of polyphenols in the regulation of ageing hallmarks. Int. J. Mol. Sci..

[17] Tran H.T.T., Schlotz N., Schreiner M., Lamy E. (2019). Short-term dietary intervention with cooked but not raw brassica leafy vegetables increases telomerase activity in CD8+ lymphocytes in a randomized human trial. Nutrients.

[18] Chan R., Woo J., Suen E., Leung J., Tang N. (2010). Chinese tea consumption is associated with longer telomere length in elderly Chinese men. Br. J. Nutr..

[19] Kim S.Y., Lee J.H., Kang N., Kim K.N., Jeon Y.J. (2021). The effects of marine algal polyphenols, phlorotannins, on skeletal muscle growth in C2C12 muscle cells via smad and IGF-1 signaling pathways. Mar. Drugs.

[20] Chen X., Le Y., Tang S.Q., He W.Y., He J., Wang Y.H., Wang H.B. (2022). Painful Diabetic Neuropathy Is Associated with Compromised Microglial IGF-1 Signaling Which Can Be Rescued by Green Tea Polyphenol EGCG in Mice. Oxidative Med. Cell. Longev..

[21] Tatari M., Jadidi E., Shahmansouri E. (2020). Study of some physiological responses of different pomegranate (*Punica granatum* L.) cultivars under drought stress to screen for drought tolerance. Int. J. Fruit Sci..

[22] Tinebra I., Scuderi D., Sortino G., Mazzaglia A., Farina V. (2021). Pomegranate cultivation in Mediterranean climate: Plant adaptation and fruit quality of ‘Mollar de Elche’ and ‘Wonderful’ cultivars. Agronomy.

[23] Alshinnawy A.S., El-Sayed W.M., Sayed A.A., Salem A.M., Taha A.M. (2021). Telomerase activator-65 and pomegranate peel improved the health status of the liver in aged rats; multi-targets involved. Iran. J. Basic Med. Sci..

[24] Farhat G., Malla J., Vadher J., Al-Dujaili E.A. (2025). Effects of Pomegranate Extract on Inflammatory Markers and Cardiometabolic Risk Factors in Adults Aged 55–70 Years: A Randomised Controlled Parallel Trial. Nutrients.

[25] Farhat G., Malla J., Al-Dujaili E.A., Vadher J., Nayak P., Drinkwater K. (2025). Impact of Pomegranate Extract Supplementation on Physical and Cognitive Function in Community-Dwelling Older Adults Aged 55–70 Years: A Randomised Double-Blind Clinical Trial. Geriatrics.

[26] WHO https://www.who.int/publications/i/item/9789240002654.

[27] Ainsworth B.E., Haskell W.L., Herrmann S.D., Meckes N., Bassett D.R., Tudor-Locke C., Greer J.L., Vezina J., Whitt-Glover M.C., Leon A.S. (2011). 2011 Compendium of Physical Activities: A second update of codes and MET values. Med. Sci. Sports Exerc..

[28] O’Callaghan N.J., Fenech M. (2011). A quantitative PCR method for measuring absolute telomere length. Biol. Proced. Online.

[29] Vitale G., Pellegrino G., Vollery M., Hofland L.J. (2019). ROLE of IGF-1 system in the modulation of longevity: Controversies and new insights from a centenarians’ perspective. Front. Endocrinol..

[30] Chen L.Y., Wu Y.H., Liu L.K., Lee W.J., Hwang A.C., Peng L.N., Lin M.-H., Chen L.K. (2018). Association among serum insulin-like growth factor-1, frailty, muscle mass, bone mineral density, and physical performance among community-dwelling middle-aged and older adults in Taiwan. Rejuvenation Res..

[31] Handayaningsih A.E., Iguchi G., Fukuoka H., Nishizawa H., Takahashi M., Yamamoto M., Herningtyas E.-H., Okimura Y., Kaji H., Chihara K. (2011). Reactive oxygen species play an essential role in IGF-I signaling and IGF-I-induced myocyte hypertrophy in C2C12 myocytes. Endocrinology.

[32] Alberti C., Chevenne D., Mercat I., Josserand E., Armoogum-Boizeau P., Tichet J., Léger J. (2011). Serum concentrations of insulin-like growth factor (IGF)-1 and IGF binding protein-3 (IGFBP-3), IGF-1/IGFBP-3 ratio, and markers of bone turnover: Reference values for French children and adolescents and z-score comparability with other references. Clin. Chem..

[33] Ammar A., Turki M., Chtourou H., Hammouda O., Trabelsi K., Kallel C., Abdelkarim O., Hoekelmann A., Bouaziz M., Ayadi F. (2016). Pomegranate supplementation accelerates recovery of muscle damage and soreness and inflammatory markers after a weightlifting training session. PLoS ONE.

[34] Ammar A., Bailey S.J., Chtourou H., Trabelsi K., Turki M., Hökelmann A., Souissi N. (2018). Effects of pomegranate supplementation on exercise performance and post-exercise recovery in healthy adults: A systematic review. Br. J. Nutr..

[35] Bachagol D., Joseph G.S., Ellur G., Patel K., Aruna P., Mittal M., China S.P., Singh R.P., Sharan K. (2018). Stimulation of liver IGF-1 expression promotes peak bone mass achievement in growing rats: A study with pomegranate seed oil. J. Nutr. Biochem..

[36] Sheng R., Gu Z.L., Xie M.L. (2013). Epigallocatechin gallate, the major component of polyphenols in green tea, inhibits telomere attrition mediated cardiomyocyte apoptosis in cardiac hypertrophy. Int. J. Cardiol..

[37] D’Angelo S. (2023). Diet and aging: The role of polyphenol-rich diets in slow down the shortening of telomeres: A review. Antioxidants.

[38] Queen B.L., Tollefsbol T.O. (2010). Polyphenols and aging. Curr. Aging Sci..

[39] Epel E. (2012). How “reversible” is telomeric aging?. Cancer Prev. Res..

