# Supplementary material for: Effects of Pomegranate Extract on IGF-1 Levels and Telomere Length in Older Adults (55–70 Years): Findings from a Randomised Double-Blinded Controlled Trial
Source: Nutrients. 2025 Sep 16;17(18):2974. doi: 10.3390/nu17182974 (PMC12472374; doi:10.3390/nu17182974)
Supplement: Supplementary file 1 [file nutrients-17-02974-s001.zip › nutrients-3861795-supplementary.pdf]

Supplementary material S1: Changes in Energy and macronutrient intake across three time points

Variable	Baseline (n=72)	Week 6 (n=70)	Week 12 (n=66)
**Energy (Kcal)**	1717 (401)	1686 (371)	1702 (433)
**Carbohydrate (g)**	187 (43)	184 (48)	185 (53)
**Protein (g)**	75 (50)	69 (18)	70 (19)
**Fat (g)**	69 (22)	69 (20)	70 (25)

Values are presented as mean (SD). No significant changes between the 3 time points were noted ($p>0.05$).
